# Three cases of hemoglobin M disease in a family lineage: Case report and literature review

**DOI:** 10.1097/MD.0000000000040652

**Published:** 2025-01-24

**Authors:** Yuhong Shen, Yue Wang, Lu Xue, Chunhuai Li, Lili He, Shuangshuang Wu

**Affiliations:** a Department of Pediatric Hematology, Children’s Medical Center, The First Hospital of Jilin University, Changchun, China.

**Keywords:** cyanosis, diagnosis, ethemoglobinemia, gene, hemoglobin M

## Abstract

**Rationale:**

This study presents a case of hemoglobin M disease (HMD), a rare inherited disorder characterized by persistent cyanosis and hypoxemia, observed across 3 generations within a single family. The diagnosis of HMD poses significant challenges, particularly in asymptomatic individuals, due to its rarity and the subtlety of its symptoms. Notably, there is a scarcity of reports on methemoglobinemia in pediatric populations, which further complicates early detection and intervention. The primary objectives of this study are to enhance awareness of HMD, to advocate for accurate diagnosis and timely treatment, and to underscore the necessity of considering this condition in patients with a familial history of cyanosis.

**Patient concerns:**

A 6-year-old boy presented to our hospital with fatigue and cyanosis in his lips and mouth. Both the child’s mother and grandfather had a history of similar symptoms since their childhood.

**Diagnoses:**

Diagnosis of hemoglobin M disease.

**Interventions:**

The patient was treated with vitamin C.

**Outcomes:**

Genetic testing revealed that the patient and her mother carried the c.190C > T mutation, leading to the p.H63Y amino acid change linked to the methemoglobin (MetHb) mutation. Despite undergoing vitamin C therapy, the patient’s symptoms of cyanosis and fatigue persisted.

**Lessons:**

Hemoglobin M disease can be readily diagnosed with a delay. Methemoglobinemia should be considered in patients with a family history of cyanosis but no cardiopulmonary disease. Hospital blood gas analysis should include a protocol for detecting methemoglobin, and genetic testing can help confirm the diagnosis.

## 1. Introduction

Methemoglobinemia, an uncommon disorder characterized by cyanosis and hypoxemia, poses challenges in diagnosis and treatment. Symptomatic cases are often misdiagnosed or overlooked, while asymptomatic presentations are even harder to identify. The classification of methemoglobinemia as acquired or hereditary depends on the underlying cause. Acquired methemoglobinemia, typically resulting from drug ingestion or toxic exposure, involves the oxidation of divalent iron in hemoglobin to trivalent iron, forming methemoglobin (MetHb). In contrast, hereditary MetHb is due to an autosomal recessive variant of the CYB5R3 gene or an autosomal dominant variant of the pearl protein gene, collectively called hemoglobin M disorder (Hb M). It is mainly characterized by cyanosis since childhood without a history of cardiopulmonary disease. For example, a boy diagnosed with hemoglobin M at age 6 inherited the condition due to a gene mutation altering the exon 190 coding. This mutation (C–A at nucleotide 190) resulted in an amino acid change (p.H63Y) from histidine to tyrosine at position 63. By highlighting such cases, we aim to increase clinician awareness and advocate for routine reporting of MetHb levels in blood gas analyses to enhance diagnostic accuracy.

## 2. Case description

The boy presented to our hospital with cyanosis of the lips and mouth that had persisted for 6 years, along with recent weakness for 7 days. The child had a normal birth history and showed no signs of growth retardation. The parents were not consanguineous. Notably, both the child’s mother and maternal grandfather exhibited similar clinical manifestations. Upon physical examination, the child’s facial color was slightly dark, with evident dark nail beds in the fingers and metatarsals. No clubbing or rash was observed on the body, and there were no signs of yellowish scleral staining. Additionally, there were no abnormalities detected in the superficial lymph nodes, liver, spleen, heart, lungs, or neurological system. Laboratory tests revealed a routine blood testing of white blood cells (WBC) at 6.82 × 10^9^/L, with 94% neutrophils and 5% lymphocytes. Red blood cell count (RBC) was 3.51 × 10^12^/L, with a corresponding hemoglobin level of 101 g/L. Further analyses showed a mean corpuscular volume of 90.6 fL, mean corpuscular hemoglobin volume of 28.8 pg, and mean corpuscular hemoglobin concentration (MCHC) of 318 g/L. Platelet count (PLT) was 353 × 10^9^/L. No abnormalities were detected in iron metabolism, folic acid, vitamin B12 levels, or hemolysis tests. Normal results were also found in coagulation studies, liver and kidney function tests, cardiac enzymes, and urine and stool analyses. Interestingly, the blood color appeared dark chocolate at the time of collection. Transcutaneous oxygen saturation was notably low at 60% to 70%. Blood gas analysis from venous blood revealed a pH of 7.37, pCO_2_ of 40 mm Hg, and pO_2_ of 60 mm Hg, with other parameters approximately within normal ranges. Arterial blood gas analysis indicated a pH of 7.45, pCO_2_ of 30 mmHg, and pO_2_ of 90 mmHg. Moreover, bone marrow cytomorphology displayed an increased proportion of erythrocytes at 59.5%, predominantly middle and late-stage erythrocytes, along with some binucleated erythrocytes. Iron staining revealed 3 + external iron, 74% internal iron, and the absence of cyclical iron patterns. Additional investigations, including a dynamic electrocardiogram, cardiac ultrasound, and abdominal ultrasound, showed no abnormalities (see Table 1).

**Table 1:** Clinical feature of the case.

Variable	Normal range	Result
Hemoglobin (g/L)	130–175	101
MCV (fL)	82–100	90.6
MCH (pg)	27–34	28.8
Reticulocyte (%)	0.67–1.90	5.93
Iron metabolism	Normal	Normal
Oxygen saturation (%)	95–100	60–70
Arterial PO_2_ (mm Hg)	95–100	90
External iron	+ to ++	3+
Internal iron (%)	12–44	74

## 3. Diagnosis and treatment

**Figure 1.**
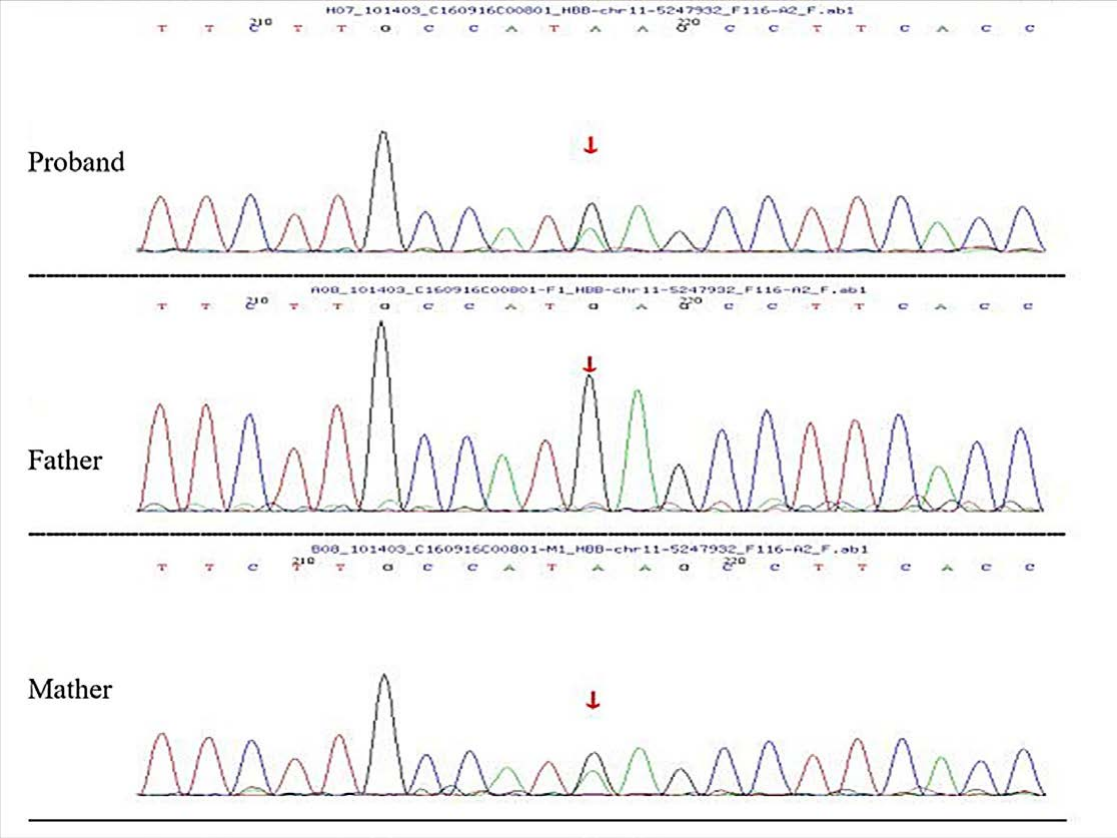
Family pedigree showing segregation of c.190C > T variants. Individuals carrying the causative gene are indicated by black circles (females) or squares (males). The filled black symbols represent the affected members, and the arrow denotes the proband.

With the informed consent of the parents and the approval of the Medical Ethics Committee, peripheral venous blood was collected from the child and his parents for genetic testing. The results indicated that both the child and his mother shared a specific genetic mutation, c.190C > T, which resulted in the amino acid change p.H63Y in the 63th amino acid and is known as Hb M-Saskatoon in the databases (see Fig. 1). This mutation is a missense mutation known to be associated with MetHb mutation^[1]^ and is reported to occur infrequently in the population. Consequently, the clinical presentation of the child, which could not be attributed to common conditions like nutritional anemia, prompted consideration of a hereditary disease, given the history of cyanosis in the child’s mother and grandfather since childhood. Family validation analysis revealed that the father did not possess the mutation, while the mother was found to be heterozygous for the locus, indicating that the child’s heterozygous mutation originated from the mother.

The clinical diagnosis of hereditary methemoglobinemia was made based on the child’s medical history, family history, clinical manifestations, and auxiliary examinations, with particular emphasis on the results of genetic examination. At follow-up, the child still has cyanosis of the lips and darkening of the face, which is more pronounced after activity but does not interfere with daily life.

## 4. Discussion

HbM is a rare autosomal dominant disorder caused by autosomal dominant mutations in the genes encoding alpha- bronchiolin (HBA1, HBA2) and beta-bronchiolin (HBB), as well as gamma-bronchiolin (HBG1, HBG2).^[2]^ In HbM, structural abnormalities of the bronchiolin portion of the protein result in spontaneous oxidation of the Fe^2+^ embedded in the hemoglobin, maintaining the Fe^3+^ state.^[3]^ The primary clinical manifestation is cyanosis, which may be the sole presenting symptom.^[4]^ In this case, the child was born with cyanosis of the lips and mouth, and the child’s mother and maternal grandfather also had a history of cyanosis since childhood. Genetic testing revealed that both the child and the mother had heterozygous mutations at this locus, indicating that they were carriers of the HbM mutation. Unfortunately, the child’s maternal grandfather did not undergo genetic testing. However, he exhibited the same clinical manifestations, indicating that this is a rare case of 3 cases of HbM in 1 family.

In this study, the children presented with cyanosis from an early age, and the degree of cyanosis was not related to respiration. His blood was dark chocolate and failed to change to red after 15 minutes of exposure to air, but changed to bright red with the addition of a reducing agent. The children’s arterial and venous blood showed normal levels of partial pressure of oxygen and oxygen saturation. Notably, oxygen delivery failed to relieve cyanosis, and cardiopulmonary examination was unremarkable. Consequently, a preliminary diagnosis of methemoglobinemia was established. Further evaluation is required to ascertain whether the condition is acquired or hereditary. Presenting similar clinical features to the child, both the child’s mother (aged 31) and maternal grandfather (aged 62) showed signs of a gloomy complexion from childhood, accompanied by weakness following strenuous activity. Notably, both maintained normal function and displayed cyanosis on the lips and nail beds without any congenital cardiovascular, respiratory, or neurological ailments. The mutation of the 190th nucleotide from cytosine to thymine resulted in the amino acid substitution p.H63Y, impacting the 63th amino acid from histidine to tyrosine. This missense mutation, identified as heterozygous and originating from the mother, is rarely found in the population and is associated with the MetHb variant. Family validation confirmed the absence of the variation in the child’s father but the presence of a heterozygous status in the mother, linking the clinical manifestations of methemoglobinemia to this locus, signifying a pathogenic variant. Therefore, both the child and the mother are diagnosed with methemoglobinemia. While the child’s maternal grandfather exhibits similar symptoms, his noncompliance prevented the collection of a blood sample. Though gene sequencing was unavailable for the paternal grandfather, this lineage is suspected to carry the methemoglobinemia gene, necessitating further analysis to determine the specific variant involved.

At least 13 different HbM variants have been reported. They are named HbM-Boston, Iwate, Saska-toon, and Hyde Park after the geographic name in which they were discovered.^[2]^ Similarly, mutations in the HbF γ chain have been reported and named Hb F-M-Osaka^[5]^ and Hb F-M-Fort Ripley,^[6]^ among others. HbM has a variable onset and duration of cyanosis due to the nature of the pearin mutant chain. Alpha-pearl proteins are associated with life, and their variants may cause cyanosis at birth. In contrast, the clinical manifestations caused by beta-pearl protein variants become apparent only after the beta-chain replaces the fetal gamma-chain at 6 to 9 months of age. The gamma-pearl protein variant mainly manifests as bruising after birth, and the bruising usually resolves spontaneously after 5 to 6 months of life as Hb F is gradually replaced by adult hemoglobin. The gene sequencing result for our child revealed a mutation at amino acid position 63 (c.190C > T), changing histidine to tyrosine. This mutation is in the beta-pearl protein gene, resembling the Hb-M Saskatoon mutation. Heterozygotes of this disease exhibited persistent cyanosis, with A few intermittent episodes observed, and the tolerance to exercise was close to normal. Their lifespan was no different from that of the general population, and the activities of Cytb5 and NADH-Cytb5R were normal. The treatment of vitamin C or methylene blue was ineffective. The activities of Cytb5 and NADH-Cytb5R were normal, vitamin C or methylene blue treatment was ineffective,^[7]^ and the prognosis was good without treatment. This child was admitted to the hospital and treated with intravenous vitamin C. Cyanosis was not relieved. It has also been suggested that both methylene blue and vitamin C therapy are ineffective in the treatment of HbM and unstable hemoglobin and should be avoided.^[8]^ Furthermore, the repeated use of methylene blue and increasing doses may result in elevated levels of methylene blue as an oxidizing agent and unchanged levels of white methylene blue as a reducing agent, thereby increasing the risk of further deterioration of methemoglobinemia.

The clinical features of methemoglobinemia are nonspecific, which increases the likelihood of misdiagnosis or delays in diagnosis and treatment. In cases where methemoglobinemia is suspected, measuring MetHb levels is a straightforward diagnostic method, and modern blood gas analyzers can accurately measure MetHb levels. Thus, routine reporting of MetHb levels in the results of blood gas analyses can aid clinicians in promptly identifying methemoglobinemia, leading to timely treatment or preventing unnecessary medication. Notably, a chocolate brown appearance of the blood sample has been associated with MetHb levels >10%,^[9]^ providing an initial visual clue for diagnosing methemoglobinemia. For instance, in the case discussed, both arterial and venous blood samples obtained from the child exhibited a dark chocolate brown color, which did not change upon exposure to air. This observation raised suspicions of elevated MetHb levels in the child. Although the initial blood gas analysis did not report these findings, a standard practice has been implemented in our outpatient and emergency clinics to routinely report MetHb levels in blood gas analyses to support emergency physicians in promptly identifying and managing such cases.

This study encountered several limitations that may have impacted the comprehensiveness of our findings. Firstly, the limited number of cases restricted our ability to conduct large-scale statistical analyses, thereby hindering our capacity to accurately determine the prevalence of the disease within the general population. Secondly, the absence of genetic samples from the patients’ maternal grandfathers posed a significant challenge, as it constrained our investigation into familial inheritance patterns and limited our understanding of potential hereditary factors. Lastly, resource constraints prevented us from conducting dynamic monitoring of all possible MetHb levels, which may have impeded our ability to fully comprehend the progression of the disease.

## 5. Conclusions

In this study, the child exhibits cyanosis from childhood and does not present with clinical signs of hemolysis. Gene sequencing has revealed that the child carries an autosomal dominant heterozygous variant, which is inherited from the mother. Furthermore, the analysis indicates that this heterozygous variant in the mother can be traced back to the child’s maternal grandfather. Through the comprehensive analysis of 3 generations of the family, Cytb5 defects and NADH-Cytb5R defects have been excluded. The occurrence of MetHb due to hemoglobin M disorder is an exceedingly uncommon condition. In cases where a child displays cyanosis from early childhood, has a family history of cyanosis, presents with dark chocolate-colored blood, shows normal arterial and venous oxygen partial pressure, and lacks cardiopulmonary disease, the possibility of methemoglobinemia should be considered. It is advisable to conduct a blood gas analysis to determine the MetHb levels and consider genetic testing for a definitive diagnosis.

## Acknowledgments

We express our gratitude to all the individuals who participated in the study.

## Author contributions

**Investigation:** Lu Xue, Lili He.

**Methodology:** Chunhuai Li.

**Writing – original draft:** Yuhong Shen, Yue Wang.

**Writing – review & editing:** Yue Wang, Shuangshuang Wu.
